# 

**Published:** 1921-04-16

**Authors:** 


### Points from Annual Reports.

### Royal Waterloo Hospital.

Under the presidency of the Duchess of Albany and the chairmanship of Earl of Athlone, a Special Committee has been appointed to raise £80,000 for extensions and additions at the Royal Waterloo Hospital. About £21,000 has already been obtained.

### Paying Patients at the West London.

Adult in-patients at the West London Hospital are to be charged 21s. a week, children from 7s. to 14s., and out-patients 6d. per attendance. Free treatment will be given if, after inquiry, patients are found unable to pay these charges.

### Chelsea Hospital for Women.

Lady Ilchester, President of the Ladies' Committee, and Lord Londonderry, President of Chelsea Hospital for Women, appeal for £20,000, and announce that on May 3 they are jointly presiding at a dinner at the Hotel Cecil with the object of raising this sum.

### A Good Start at Accrington.

After six months' work the Workpeople's Hospital Fund for the benefit of Accrington Hospital records 144 contributing mills and firms. The local Tradesmen's Association has also agreed to organise the employees of its members. The income received in this first six months has amounted to £2,346.

### Hospital Appeals in Ireland.

The Dublin Hospitals last year issued a joint appeal for £100,000 to pay off their debts. They made their state known, held a joint festival, and organised collections. The result was that £35,000 was raised. On analysis we find that the fête realised £15,655, and the collections £19,000, of which residents in Dublin gave £9,000! This poor result has led to the Council of the Associated Hospitals to renew its appeal to employers and their staffs for regular contributions.

### Chesterfield Royal Hospital.

Over £35,000 has been received towards the £50,000 needed for the new out-patient department and additional accommodation for the nursing, domestic, and administrative staff. The income was well maintained last year, and there was an adverse balance of only £1,552 despite the huge expenditure of £38,535.

### Medical Appointments.

Dr. A. A. Cockayne, of Reading, has been appointed assistant schools medical officer at Newport (Mon.).

Dr. E. T. Pinkey has been appointed medical officer of the City of London at a salary of £900 per annum, with unfurnished residence free of rates and taxes.

### Passing Events and Topics.

### Schoolgirls' Help for a Hospital.

The pupils of the Tollington High School for Girls have collected during the present term the sum of £50 7s. 6d. in aid of the Great Northern Hospital. The school has "adopted" the hospital and maintains a bed in one of the general wards.

### The Great Northern and Insured Patients.

The Committee of Management of the Great Northern Central Hospital have placed on record their opinion that insured persons treated both as in- and out-patients should be paid for in full by approved societies by means of capital grants, and not by the handing over of lump sums.

### Adoption of Hospitals.

In reference to the "Adopt a Hospital" scheme, we are informed that the staff of Bovril, Ltd., have for a considerable time contributed to the funds of St. Bartholomew's, and have undertaken to collect not less than £100 in the coming twelve months.

### Bristol Infant Welfare Association.

A Special Course of lectures on "Infant Care" for mothers and all who are interested in Infant Welfare will be held at the Folk House, College Green, Bristol, on Wednesdays, from April 6 to May 4, 1921, inclusive. The lecturers are Dr. Muriel Radford, Dr. Eric Pritchard, Dr. David Forsyth, Dr. H. C. Cameron, Mr. C. Peyton Baly, and Miss Margaret McMillan. Further details may be obtained from Mrs. Wethered, Hon. Secretary, Bristol Infant Welfare Association, 11 The Avenue, Clifton; or from Miss Halford, Secretary, National Association for the Prevention of Infant Mortality, 4 and 5 Tavistock Square, London, W.C. 1.

### R.A.M.C. War Memorial.

Up to the present the Royal Army Medical Corps Memorial Fund totals £19,328, of which £1,566 has been earmarked for families and dependants of the fallen. The memorial itself will be a tablet in Westminster Abbey, near to the grave of the Unknown Warrior, and replicas will be presented to Edinburgh and Dublin to be placed in positions chosen by the city authorities.

### The Ex-Services Welfare Society.

The first Recuperative Home under the management of this Society has been opened at Chartfield, 50 Putney Hill, S.W. 15, the honorary matron-superintendent being Mrs. G. M. Clarke. In our issue of March 5, page 522, we gave a brief account of the objects. The Homes are for all ranks of all branches of His Majesty's Forces suffering from the more acute forms of neurasthenia and mental breakdown. The Society, which is in co-operation with the Veterans' and Officers' Associations, is registered under the War Charities Act, 1916, and has its offices at 20 Craven Road, Hyde Park, the honorary organiser being Mrs. G. M. Clarke.

### Parliamentary Medical Committee.

### DR. ADDISON'S SERVICES.

The Medical Committee met on Monday, April 11, at the House of Commons. The following resolution was put and passed unanimously: That the medical members of the House of Commons express their cordial appreciation of the services of the Right Hon. Christopher Addison, M.P., as Minister of Health, and congratulate him on the excellent work he has done as the first Minister in the face of many difficulties. They wish to place on record their admiration for his zeal and devotion to duty and his untiring work in the interests of public health.

The Public Health Officers (Security of Tenure) Bill was discussed, and the Committee decided to lend it their support. The Coroners Bill was considered, and it was decided that when this Bill reached Committee a new Clause should be moved to increase the fees paid for post-mortem examination and medical evidence. The Tuberculosis Bill and the Health Estimates were then considered. The Dogs Bill was discussed, and opposition to it was agreed upon unanimously.

### Matrons' and Sisters' Appointments.

Municipal Hospital, Warrington.—Miss Beatrice Evans has been appointed sister-in-charge. She was trained at the Manchester Royal Infirmary, and has held the post of sister at the City Hospital, Coventry, Guest Hospital, Dudley, Cumberland Infirmary, and at the Municipal Hospital, Willesden. She has also done military nursing.

Bolton Infirmary and Dispensary.—Miss Lucy Lees and Miss Lucy Henshall have been appointed sisters. Miss Lees was trained at Bolton and at the Ramsbottom Cottage Hospital, where she was later staff nurse. She has been sister at Blair Hospital, Bromley Cross. Miss Henshall was trained at the Hull General Infirmary, has been staff nurse at Rochdale Infirmary, served in Q.A.I.M.N.S.R., and sister at the South Devon Hospital, Plymouth, at Merthyr Hospital, and at the West Kent Hospital, Maidstone.

Maternity Home, Stockport.—Miss Bessie Louisa Scott has been appointed matron. She was trained at Shirley Warren Infirmary, has been superintendent at Warwick Road Hospital, Banbury, night sister and assistant superintendent at Stow Hill Hospital, Newport (Mon.), children's and maternity sister at Steyning Infirmary, Brighton, and at present is superintendent of nurses at Basford and Bulwell Infirmary, Nottingham.

Cossham Memorial Hospital, Kingswood, Bristol.—Miss Mabel R. Briggs has been appointed night sister. She was trained at the Infirmary, Sculcoates, Hull, and has been ward sister at the Shropshire Orthopædic Hospital.

Tranmere Infirmary, Birkenhead.—Miss Sophia Smith Bevan, A.R.R.C., has been appointed matron. She was trained at Guy's Hospital, and holds the certificates of the Central Midwives Board and the Chartered Society of Massage and Medical Gymnastics. She has been sister, second and first assistant matron at Fulham Infirmary.

Clayton Hospital, Wakefield.—Miss Agnes Cameron has been appointed matron. She was trained at Leeds General Infirmary, where she has held the posts of night superintendent and second assistant matron.

London Homœopathic Hospital, Great Ormond Street, W.C.—Miss J. Scorgie, R.R.C., and Miss E. England have been appointed sisters. Miss Scorgie was trained at Manchester Royal Infirmary, where she was later theatre sister and ward sister; she was sister in the Scottish Women's Hospital, Serbia, and for four years served in the Q.A.I.M.N.S.R. Miss England was trained at the Royal City of Dublin Hospital, where she was later night sister and housekeeping sister.

Royal Mineral Water Hospital, Bath.—Miss M. K. Dietz and Miss Fanny Roberts have been appointed night and senior sisters respectively. Miss Dietz was trained at Radcliffe Infirmary, Oxford, where she has been sister of the women's surgical ward and of the theatre and out-patients; she has held the post of senior sister and home sister at the Royal Surrey County Hospital, Guildford, ward sister and assistant lady superintendent at St. George's European General Hospital, Bombay, and has served in the Q.A.I.M.N.S.R. at the Connaught Military Hospital, Aldershot. Miss Roberts was trained at Cheltenham General Hospital, where she was later theatre sister and surgical sister, and has been home sister and night sister at the Royal Berkshire Hospital, Reading, and assistant matron of the Cumberland Infirmary, Carlisle.

THE

### COLLEGE OF NURSING

(Limited by Guarantee.)

### What the Local Centres are Doing.

### LOCAL CENTRES FOR HAMPSHIRE.

At a meeting recently held at the Royal South Hants and Southampton Hospital, presided over by Miss Jenkins, the Matron, it was decided to form a Centre in Southampton. Those desiring to join should communicate with Miss Jenkins as above, marking their envelope "College of Nursing." A meeting will be held on April 21, at 3 p.m. to elect officers and committee.

Following on a meeting at the Royal Portsmouth Hospital, a Centre has been formed for Portsmouth. Miss Alcock has been nominated Chairman, Miss Duffy Hon. Treasurer, and the Hon. Felicie Norton Hon. Secretary.

### NORFOLK AND NORWICH CENTRE.

(Hon. Secretary, Miss V. A. Rutter, 43 Newmarket Road.)

Thursday, April 21, 8 p.m.—At the Norfolk and Norwich Hospital, lecture on "Travels," by Sir Eustace Gurney. The Representative Committee will meet at 7.15 p.m. before the lecture. Tuesday, April 26, 4.30 p.m., Annual Meeting at the Norfolk and Norwich Hospital.

### YORKSHIRE CENTRE, LEEDS.

(Hon. Secretary, Miss Lindall, Hospital for Women and Children.)

On Monday, March 31, between sixty and seventy members spent a most enjoyable evening at a whist-drive, held in the nurses' home of the General Infirmary, Leeds, when Miss Innes, R.R.C., was hostess.

Thursday, April 21, 6 p.m.—Annual Meeting at Collinson's Café, Albion Street, Leeds. Business, election of officers and members of Committee for the ensuing year. Tea will be provided. Members are reminded that their annual subscription, 5s., is due.

Theatre Parties.

Two theatre parties, at the Grand Theatre, Leeds, have been arranged, one for the week commencing May 23, "Henry Baynton," and another for the week commencing May 30, "The Skin Game." The Centre will pay half the price of the booked seats. Members wishing to join should let the Hon. Secretary know not later than April 21 stating which week they wish to go.

### Personalia.

On March 28, at St. Philip's Church, Litherland, by the Rev. J. Howard Preston, B.Sc., Arnold Bennett, M.P.S., dispenser at the Ministry of Pensions Hospital, Maghull, was married to Alice Rothwell, staff nurse, late of Q.A.I.M.N.S.R. and Ministry of Pensions Nursing Service, of Litherland, Liverpool.

The Earl of Arran, P.C., K.P., Chairman of the Royal Westminster Ophthalmic Hospital, and Mr. Gustavus Hartridge, F.R.C.S., consulting surgeon, gave evidence upon the Special Hospitals, before Lord Cave's Committee, at the Ministry of Health on Wednesday, April 6.

Mrs. Linington, wife of Dr. Linington, has been awarded the Medal of Queen Elizabeth for services rendered to the Belgian refugees in the Belgian Maternity Hospital, Folkestone. This hospital was started and put into working order in less than two days. Other Folkestone ladies thus honoured are Lady Lucy Markham, Sister Mumford, of the Bevan Hospital, Sandgate, Miss Wyles, former Matron of the Royal Victoria Hospital, Mrs. Percy Lewis, Mdle. Frassen, a prominent Belgian refugee, and Mrs. G. M. Petersen, wife of the Vice-Consul in Folkestone.

### General Nursing Council for Scotland.

At the meeting of this Council, held at 13 Melville Street, Edinburgh, on April 6, eleven members were present, and in the absence of the Chairman and Vice-Chairman, Dr. H. E. Fraser was voted to the chair.

A letter was submitted from the Scottish Board of Health announcing its appointment to the Council of Miss Margaret M. White, Superintendent of Queen Victoria's Jubilee Institute for Nurses, 26 Castle Terrace, Edinburgh.

The Registrar was instructed to endeavour to arrange a meeting with the Scottish Board of Health to discuss certain points outstanding with them in regard to the Draft Rules already submitted.

Miss Gill called the attention of the meeting to a report in *The Nursing Mirror* of April 2 of a meeting of the General Nursing Council for England and Wales, held on March 24, in which it was stated that the Chairman of the English Council had announced that he had discussed with two gentlemen from Scotland and one from Ireland, representing the Scottish and Irish General Nursing Councils, the question of registration of nurses trained in one country and working in another. The Registrar was instructed to point out that this statement was inaccurate, as the Council had no knowledge of any such meeting, and no one had any authority from them to discuss such a question.

The Registrar was instructed to prepare the Report required by Section 9 of the Nurses' Registration (Scotland) Act for submission to the Scottish Board of Health on the work of the Council to December 31 last. He submitted the Report of the Government auditor on the accounts of the Council to December 31, 1920.

The next meeting of Council is fixed for April 20.

### Village and Country Town Concerts.

### A SCHEME FOR PROVINCIAL HOSPITALS.

An interesting letter from Mr. Walpole E. Sealy is published on another page, wherein he invites hospital superintendents who would welcome a free concert to communicate with him. The scheme, of which he is the honorary organiser, started in 1919 to give opportunities for hearing good music to every village likely to appreciate it. The works of the great composers, from Handel and Mozart to Chopin and Brahms, have been performed already in fifteen counties.

The concert-room, sale of tickets, transport, and hospitality are left to the local organisers of the place from which the invitation comes. In return five professional artists are chosen and sent from London, and their fees and expenses are paid out of the takings at the concerts. The necessary literature and tickets are supplied by the honorary organisers. The scheme has no office expenses and no commissions, and all the organisation, central and local, is voluntary. Every penny, therefore, goes to the provision of more concerts, and the payment of adequate fees to good professional artists, than whom no members of the community have suffered more heavily from the war and the increased cost of living. Further details of this interesting scheme may be obtained from the honorary organisers, Miss Paget, 20 Clarendon Road, London, W. 11, or Rev. Walpole E. Sealy, Fonthill, East Grinstead, Sussex.

### Forthcoming Events.

The Royal Sanitary Institute, 90 Buckingham Palace Road, S.W. 1.—Wednesday, April 27, 5.30 p.m., Colonel C. H. Melville, C.M.G., M.B., R.A.M.C., on "Some Lessons of the War."

April 16, 1921] [Price Twopence

### The Hospital

**RATES OF SUBSCRIPTION** (post free, payable in advance).

Three months, 3/3; six months, 6/6; twelve months, 13/-. (Should the cost of printing and paper as well as increased postage, necessitate alteration of these rates, the period paid for will be reduced proportionately on unexpired subscriptions.) THE HOSPITAL "Index" to Volume LXVIII., April to September 1920, is now ready, price 1/1 per copy, post free. Address The Manager, THE HOSPITAL, "The Hospital" Building, 28/29 Southampton Street, Strand, London, W.C. 2.

